# Everolimus in hormone receptor-positive metastatic breast cancer: *PIK3CA* mutation H1047R was a potential efficacy biomarker in a retrospective study

**DOI:** 10.1186/s12885-019-5668-3

**Published:** 2019-05-14

**Authors:** Zongbi Yi, Fei Ma, Binliang Liu, Xiuwen Guan, Lixi Li, Chunxiao Li, Haili Qian, Binghe Xu

**Affiliations:** 1 Department of Medical Oncology, National Cancer Center/National Clinical Research Center for Cancer/Cancer Hospital, Chinese Academy of Medical Sciences and Peking Union Medical College, No.17, Panjiayuan Nanli, Chaoyang District, Beijing, 100021 China; 2 State Key Laboratory of Molecular Oncology, National Cancer Center/National Clinical Research Center for Cancer/Cancer Hospital, Chinese Academy of Medical Sciences and Peking Union Medical College, Beijing, 100021 China

**Keywords:** Biomarkers, Breast neoplasms, ctDNA, Everolimus

## Abstract

**Background:**

Everolimus, an inhibitor of mammalian target of rapamycin (*mTOR*), has been shown to increase the efficacy of endocrine therapies in hormone receptor (HR)-positive metastatic breast cancer. However, because breast cancer is a highly heterogeneous disease, the responses of different patients to everolimus may vary. Therefore, we performed this study to better select patients who will benefit most from or be resistant to everolimus.

**Methods:**

Patients with HR-positive breast cancer who were treated with everolimus at the Cancer Hospital, Chinese Academy of Medical Sciences from February 2014 to March 2017 were enrolled in the present study. Mutations in ctDNA were assayed in 1021 tumor-related genes via gene panel target capture-based next-generation sequencing.

**Results:**

In total, 120 patients with metastatic breast cancer who were treated with everolimus were enrolled in the present study. The median progression-free survival (PFS) of all patients was 5.1 months (95% confidence interval [CI] 3.9–6.3 months). No difference in survival was observed between patients who received endocrine drugs used in previous treatment regimens and patients who did not receive these drugs (median PFS 5.2 and 5.1 months, respectively, *p* > 0.05). Additionally, we did not find any difference in outcomes between patients who had primary resistance to previously used endocrine drugs and patients who had nonprimary resistance to previous treatments (*p* > 0.05). Multivariate analysis showed that < 3 metastatic sites, < 2 lines of previous endocrine therapy, < 2 lines of previous chemotherapy, and treatment with everolimus combined with fulvestrant were associated with improved survival (*p* < 0.05). Sixteen patients underwent ctDNA analysis before everolimus treatment. The frequency of *PIK3CA* gene mutations was 62.5%, and H1047R was the most frequently detected mutation. Patients with the *PIK3CA/*H1047R mutation had longer PFS than patients with wild-type or other mutant forms of *PIK3CA*, and the median PFS in these two groups of patients was 8.8 and 4.1 months, respectively (*p* < 0.05).

**Conclusions:**

Our data suggest that patients who receive more lines of chemotherapy or endocrine therapy are less likely to benefit from everolimus. For everolimus combination therapy, we can even select endocrine drugs that gave rise to primary resistance in previous treatments. Additionally, the *PIK3CA/*H1047R mutation may be a potential biomarker of sensitivity to everolimus.

**Electronic supplementary material:**

The online version of this article (10.1186/s12885-019-5668-3) contains supplementary material, which is available to authorized users.

## Background

Hormone receptors (HRs) including estrogen receptor (ER) and/or progesterone receptor are expressed in approximately 70% of breast cancers. Endocrine therapy is a very important treatment for patients with HR-positive advanced breast cancer. However, resistance to endocrine therapy is an immense challenge in clinical settings [1, 2]. One mechanism of drug resistance to endocrine therapy is the activation of the phosphatidylinositol 3-kinase (*PI3K*)/*AKT*/mammalian target of rapamycin (*mTOR*) pathway [3]. The inhibitors of the *PI3K/AKT/mTOR* pathway could reverse endocrine drug resistance [3, 4]. Everolimus is a selective inhibitor of *mTOR* that has shown potential for overcoming the resistance to endocrine therapy [5–7]. However, due to the heterogeneity of breast cancer, a subset of patients do not respond to everolimus. Therefore, it is crucial to find biomarkers that predict the efficacy of everolimus in clinical settings [8]. Several experimental studies have indicated that cancers with *PIK3CA*/*PTEN* mutations are sensitive to everolimus; however, clinical trials did not draw the same conclusions [9–16].

To better select patients who will benefit most from or be resistant to everolimus, we conducted a retrospective analysis on data from 120 patients with metastatic breast cancer who underwent therapy at the National Cancer Center/Cancer Hospital, Chinese Academy of Medical Sciences and Peking Union Medical College from February 2014 to March 2017. We also performed circulating tumor DNA (ctDNA) analysis on sixteen patients to determine the association between gene mutations and response to everolimus.

## Methods

### Patients and sample collection

Patients with HR-positive breast cancer who were treated with everolimus at the Cancer Hospital, Chinese Academy of Medical Sciences from February 2014 to March 2017 were enrolled in the present study. The following data were collected for each patient: age, nuclear grade, pathological type, ER, progesterone receptor, human epidermal growth factor receptor (HER2) status, number of metastatic sites, visceral metastases, previous treatment, treatment details and clinical course. Peripheral blood samples were collected from the patients who consented to participate in the ctDNA analysis.

This study was reviewed and approved by the Ethics Committee of the National Cancer Center/Cancer Hospital, Chinese Academy of Medical Sciences and Peking Union Medical College. This study was performed in accordance with the Good Clinical Practice guidelines and the Declaration of Helsinki. The need for informed consent from patients who did not participate in the ctDNA analysis was waived under the approval of the institutional review board due to the retrospective study design. Written informed consent was obtained from the patients who participated in the ctDNA analysis (ref: 16–038/1117).

Adverse events (AEs) were evaluated through reexamination or telephone follow-up at least once each month. We retrospectively collected information on AEs from patients’ medical records and laboratory test results. AEs were evaluated based on the National Cancer Institute Common Terminology Criteria for Adverse Events version 4.0.

### Treatment

Patients received everolimus at a dose of 10 mg/day plus endocrine therapy including exemestane, letrozole, anastrozole, fulvestrant, tamoxifen and toremifene. The dose was reduced to 5 mg/day for patients who could not tolerate 10 mg/day. Each patient also used an oral care package that prevented stomatitis. The oral care package included kangfuxinye, a pure Chinese herbal medicine extracted from the American cockroach, a special toothbrush and a user manual for the mTOR inhibitor. Treatment with everolimus was interrupted when intolerable toxicity emerged or if patients withdrew from the study. To evaluate treatment responses, computed tomography (CT) or magnetic resonance imaging (MRI) was performed every two months or whenever signs or symptoms that indicated disease progression according to Response Evaluation Criteria in Solid Tumors (RECIST) v. 1.1 were present [17].

### ctDNA analysis

Peripheral blood samples were collected in Streck tubes (Streck, Omaha, NE, USA) and were centrifuged within 72 h to separate the plasma from the peripheral blood cells. QIAamp Circulating Nucleic Acid Kits (Qiagen, Hilden, Germany) were used to extract the circulating DNA (cDNA) from 0.5–2.0 mL of the plasma samples. QIAamp DNA Blood Mini Kits (Qiagen, Hilden, Germany) were used to extract genomic DNA (gDNA) from the peripheral blood cells. Both DNA extractions were performed according to the manufacturer’s protocols, and gDNA was sequenced as the normal control sample. DNA concentration was measured using a Qubit fluorometer and the Qubit dsDNA HS (High Sensitivity) Assay Kit (Invitrogen, Carlsbad, CA, USA). The size distribution of the cfDNA was assessed using an Agilent 2100 BioAnalyzer and a DNA HS kit (Agilent Technologies, Santa Clara, CA, USA) [18]. A panel of 1021 genes was assayed in the present study (Additional file 1: Table S1). cDNA and gDNA preparation, library construction, hybrid capture, and sequencing were previously described [19]. Low-quality reads and terminal adaptor sequences were filtered out of the raw data. BWA (version 0.7.12-r1039) was employed to align the clean reads to the reference human genome (hg19). Picard (version 1.98) was used to mark PCR duplicates. GATK (version 3.4–46-gbc02625) was used for realignment and recalibration. Single nucleotide variants (SNVs) were called using MuTect (version 1.1.4) and NChot [20], a software developed in-house to review hot spot variants. GATK was used to identify small insertions and deletions (indels). CONTRA (v2.0.8) was used to identify somatic copy number variants (CNVs). Significant copy number variation was expressed as the ratio of the adjusted depth between the ctDNA and the control gDNA. We verified all the final candidate variants in the Integrative Genomics Viewer (IGV).

### Statistical analysis

PFS was calculated from the date of sample collection to the date of disease progression or death from any cause. Cases that did not reach an end point (progression or death events) were censored at the date of last follow-up. Kaplan-Meier survival plots were generated based on previous treatment or gene mutations, and curves were compared using log-rank tests. All statistical tests used in the present study were two-sided, and *p*-values below 0.05 were considered significant. All statistical analyses were performed using SPSS version 19.0 (Chicago, IL, USA).

## Results

### Patients

In total, 120 patients with metastatic breast cancer who were treated with everolimus combined with other endocrine therapies at the National Cancer Center/Cancer Hospital, Chinese Academy of Medical Sciences and Peking Union Medical College from February 2014 to March 2017 were enrolled in the present study. All patients enrolled in this study were diagnosed with HR-positive breast cancer; of those, 16 (13.3%) patients had HER2-positive breast cancer, and 104 (86.7%) patients had HER2-negative breast cancer. Nineteen (15.8%) patients received tamoxifen or toremifene plus everolimus; 18 (15%) patients received fulvestrant plus everolimus; 24 (20.0%) patients received a nonsteroidal aromatase inhibitor (AI) including letrozole or anastrozole plus everolimus; and 59 (49.2%) patients received exemestane plus everolimus. The median age was 52.5 years (range, 24 to 84 years). Fifty-four (45.0%) patients had visceral metastases, and 66 (55.0%) had nonvisceral metastases. Ninety-eight (81.7%) patients had received > 2 lines of previous endocrine therapies. The main clinical characteristics of all patients in the present study are outlined in Table 1.

**Table 1:** Population characteristics

Characteristics	All	Combined with previously used drugs		
		Primary resistance	Secondary resistance	None
All patients	120	19 (16%)	20 (17%)	81 (67%)
Mean age/years (range)	52.6 (24–84)	53.4 (31–76)	54.9 (25–75)	51.9 (24–84)
Nuclear grade, No. (%)				
1	4 (3%)	1 (5%)	0 (0%)	3 (4%)
2	92 (77%)	13 (68%)	15 (75%)	64 (79%)
3	24 (20%)	5 (26%)	5 (25%)	14 (17%)
Pathological type, No. (%)				
Infiltrating ductal carcinoma	112 (93%)	19 (100%)	16 (80%)	77 (95%)
Infiltrating lobular carcinoma	8 (7%)	0 (0%)	4 (20%)	4 (5%)
HER2 status, No. (%)				
Negative	104 (87%)	18 (95%)	18 (90%)	68 (84%)
Positive	16 (13%)	1 (5%)	2 (10%)	13 (16%)
Number of metastatic sites, No. (%)				
0–2	66 (55%)	9 (47%)	10 (50%)	47 (58%)
≥3	54 (45%)	10 (53%)	10 (50%)	34 (42%)
Number of chemotherapy lines, No. (%)				
0–2	68 (57%)	11 (58%)	8 (40%)	49 (60%)
≥3	52 (43%)	8 (42%)	12 (60%)	32 (40%)
Number of endocrine therapy lines, No. (%)				
0–1	22 (18%)	1 (5%)	2 (10%)	19 (23%)
≥2	98 (82%)	18 (95%)	18 (90%)	62 (77%)
The drug combined with, No. (%)				
SERM	19 (16%)	5 (26%)	5 (25%)	9 (11%)
SERD	18 (15%)	2 (11%)	2 (10%)	14 (17%)
NSAI	24 (20%)	4 (21%)	8 (40%)	12 (15%)
EXE	59 (49%)	8 (42%)	5 (25%)	46 (57%)
Visceral metastases, No. (%)				
Yes	54 (45%)	5 (26%)	8 (40%)	41 (51%)
No	66 (55%)	14 (74%)	12 (60%)	40 (49%)

Abbreviations: *HER2* human epidermal growth factor receptor-2; *SERM*, selective estrogen receptor modulator, *SERD* selective estrogen receptor downregulator, *NSAI* nonsteroidal aromatase inhibitor, *EXE* exemestane

### Survival analysis

**Fig. 1:**
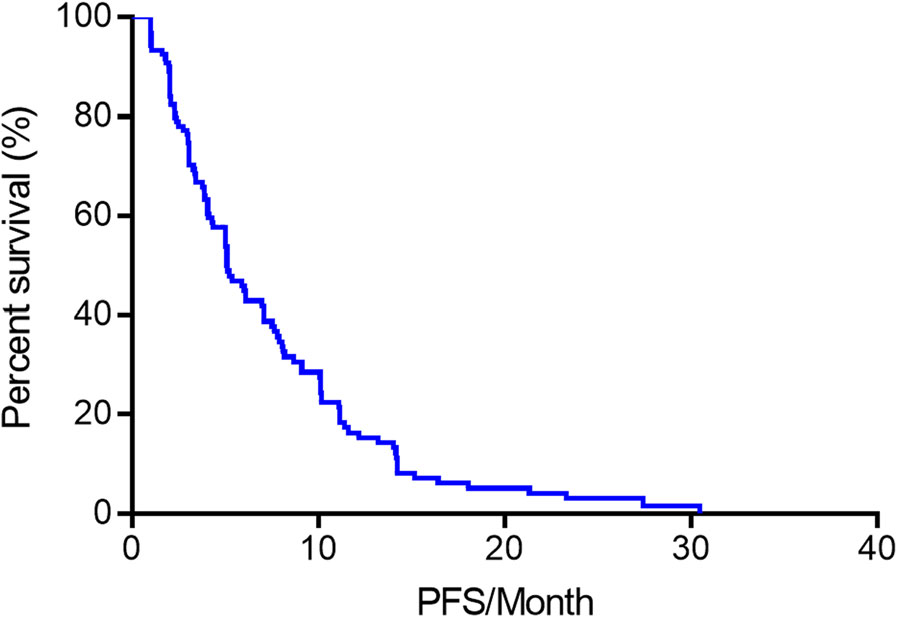
The PFS of everolimus-treated patients with HR-positive advanced breast cancer. A total of 120 patients received everolimus. PFS, progression-free survival. HR, hormone receptor

**Fig. 2:**
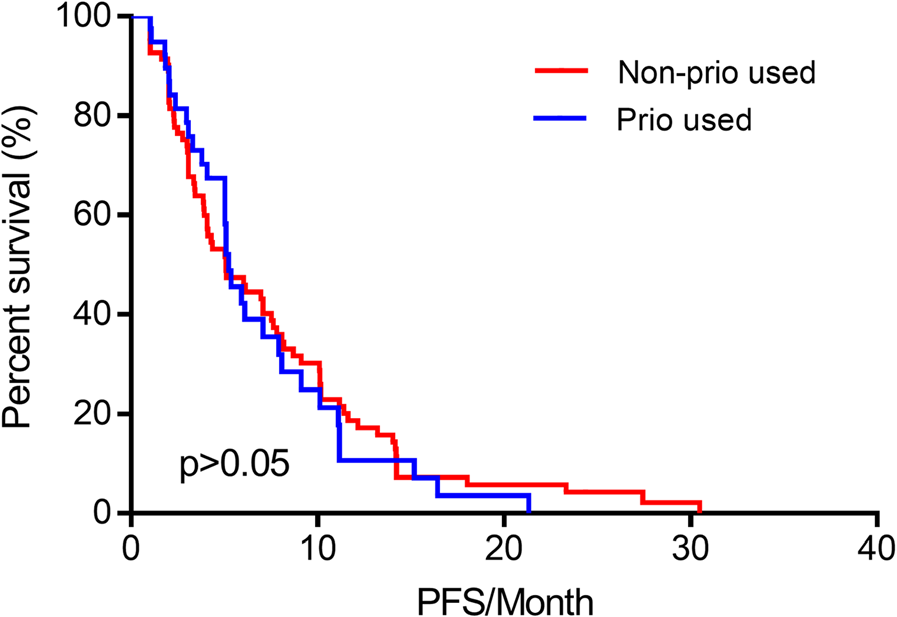
The PFS of patients treated with everolimus combined with endocrine therapy stratified by drugs that were previously used or not. A total of 120 patients received everolimus; 39 patients received a drug that was used in previous treatments (blue), and 81 patients received drugs that were not previously used (red). PFS, progression-free survival

**Fig. 3:**
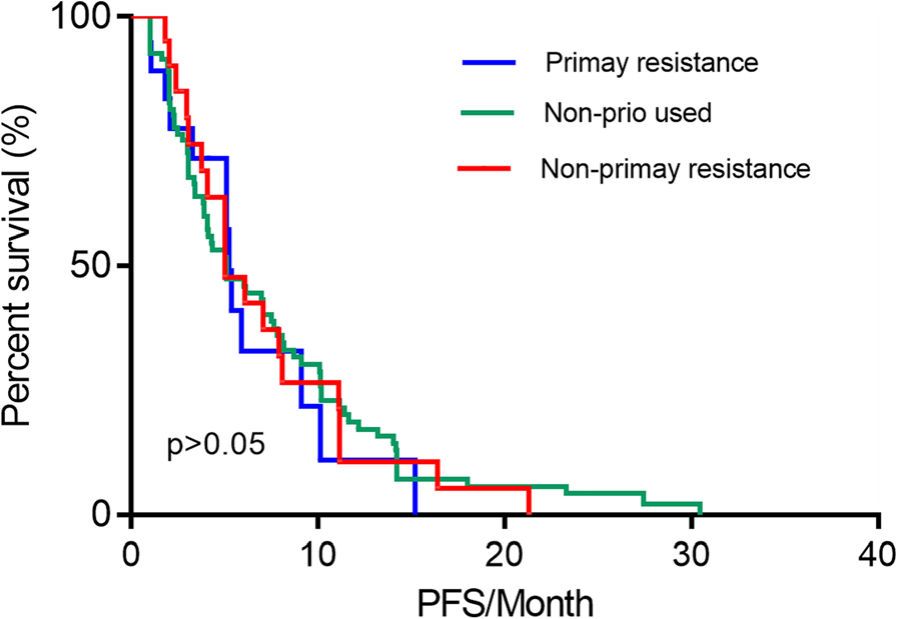
The PFS of patients treated with everolimus combined with endocrine therapy stratified by primary resistance or nonprimary resistance to drugs used in previous treatments. A total of 120 patients received everolimus; 19 patients received drugs to which they acquired primary resistance during previous treatment (blue); 20 patients received drugs to which they acquired nonprimary resistance during previous treatment (red); and 81 patients received drugs that were not previously used (green). PFS, progression-free survival

The efficacy of everolimus was evaluated in all 120 patients. None of the patients achieved complete response (CR), but partial response (PR) was observed in 19 (15.8%) patients. Additionally, 59 (49.2%) patients showed stable disease (SD) as an optimal efficacy, and 42 (35.0%) patients showed progressive disease (PD) at first assessment. The objective response rate (ORR = CR + PR) and clinical benefit rate (CBR = CR + PR + SD) in the 120 patients were 15.8 and 65.0%, respectively. The median PFS of all patients in the present study was 5.1 months (95% confidence interval [CI] 3.894–6.306 months, Fig. 1). Overall, 39 (32.5%) patients received everolimus combined with endocrine therapies that had been used in previous treatments, and 18 (46.2%) of these patients had primary resistance against those drugs. We further analyzed the relationship between PFS and previous treatments and found no difference between patients who received endocrine drugs used in previous treatment and patients who did not receive these drugs (median PFS 5.2 and 5.1 months, respectively, *p* > 0.05, Fig. 2). Additionally, we did not find any difference in outcomes between patients who had primary resistance to previously received endocrine drugs and patients who had nonprimary resistance to previous treatments (*p* > 0.05, Fig. 3).

### Safety and tolerability

All 120 patients who received everolimus treatment were eligible for safety evaluation. The grades of all major treatment-related AEs are shown in Table 2. The most common AEs were stomatitis (23.3%) and rash (8.3%). Twenty-three patients experienced grade 3 AEs, and the main grade 3 or 4 AEs were stomatitis (9.2%), increased alanine aminotransferase (ALT) or aspartate aminotransferase (AST) levels (5.0%), cough (5.0%), and anorexia (4.2%). No treatment-related death was observed in the present study.

**Table 2:** Adverse events experienced by ≥2 Patients (*n* = 120)

Adverse Event	Any Grade		Grade 3–4	
	No.	%	No.	%
Stomatitis	28	23.3	11	9.2
Rash	10	8.3	1	0.8
Increased ALT or AST	6	5.0	3	2.5
Cough	6	5.0	2	1.7
Anorexia	5	4.2	2	1.7
Asthenia	5	4.2	0	0.0
Dyspnea	4	3.3	0	0.0
Pneumonitis	3	2.5	0	0.0
Nausea	3	2.5	1	0.8
Hyperglycemia	2	1.7	0	0.0
Neutropenia	2	1.7	1	0.8
Diarrhea	2	1.7	0	0.0
Fever	2	1.7	0	0.0
Maxillary necrosis	2	1.7	0	0.0
Venous thrombosis	2	1.7	0	0.0

NOTE. Adverse events listed regardless of relationship to study drug

Abbreviations: *ALT* alanine aminotransferase, *AST* aspartate aminotransferase

### Risk factors for PFS

Based on the univariate analysis, patients treated with < 2 lines of previous endocrine therapy had better PFS than patients treated with > 2 lines of previous endocrine therapy (hazard ratio = 0.46, 95% CI 0.28–0.79, *p* = 0.005). Similarly, treatment with < 3 lines of previous chemotherapy was associated with better survival than treatment with > 3 lines of previous chemotherapy (hazard ratio = 0.48, 95% CI 0.32–0.72; *p* < 0.001). Moreover, patients with < 3 metastatic sites had better survival than patients with > 3 metastatic sites (hazard ratio = 0.41, 95% CI 0.27–0.62; *p*<0.001). In the multivariate analysis, we included the HER2 status, the number of previous endocrine therapies and chemotherapy lines, and drugs combined with everolimus. The multivariate analysis showed that improved survival was associated with < 3 metastatic sites (hazard ratio = 0.49, 95% CI 0.31–0.76; *p* = 0.002), < 2 lines of previous endocrine therapy (hazard ratio = 0.56, 95% CI 0.31–1.00, *p* = 0.048), < 2 lines of previous chemotherapy (hazard ratio = 0.59, 95% CI 0.38–0.92; *p* = 0.021), and treatment with everolimus combined with fulvestrant (hazard ratio = 0.53, 95% CI 0.28–0.99; *p* = 0.045).

### ctDNA analysis

**Fig. 4:**
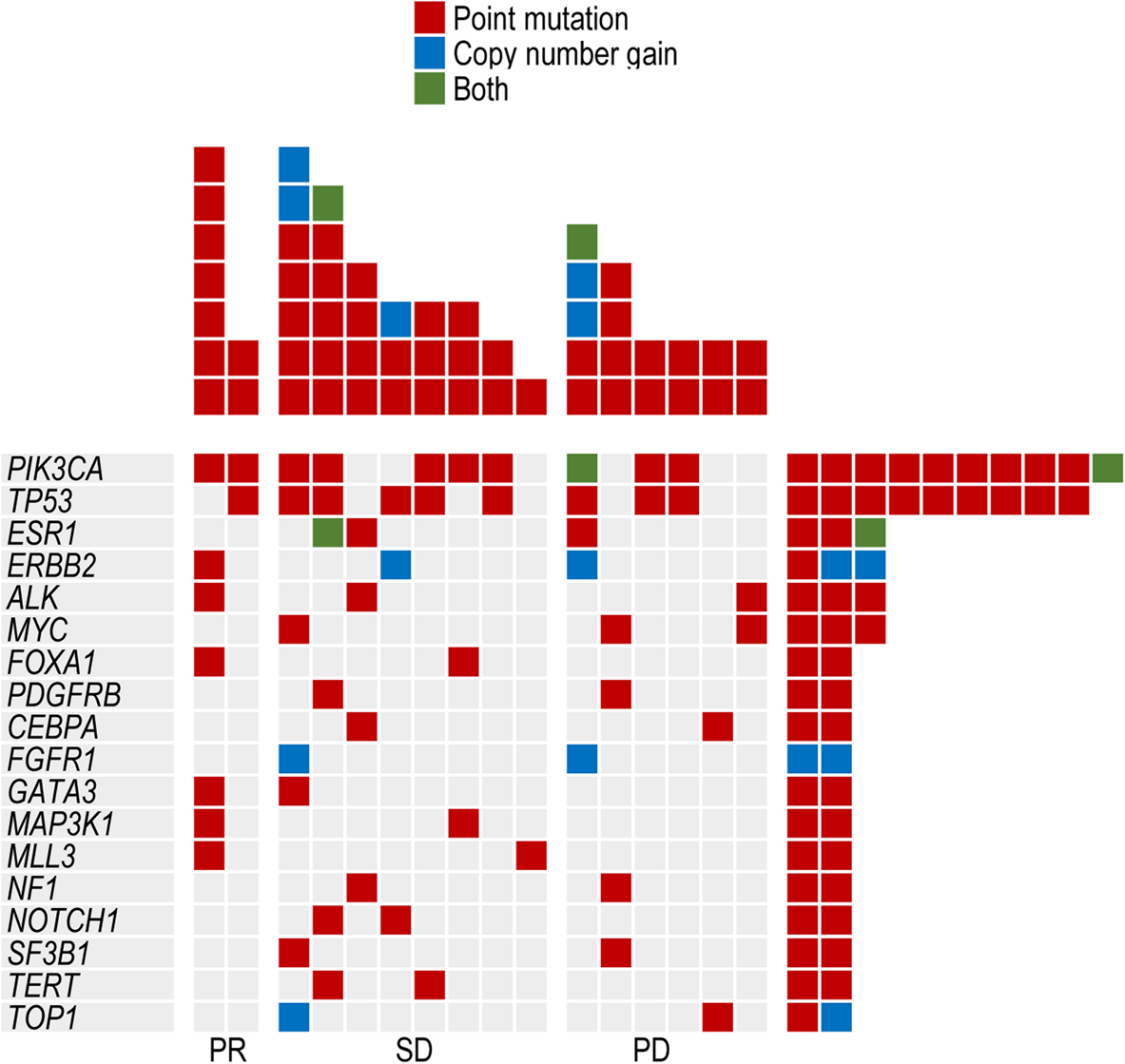
The spectrum of hotspot mutations in HR-positive breast cancer patients. Each of the 18 hotspot gene mutations has been identified in more than 2 patients listed to the left of the figure. The number of mutations in each gene among the 16 patients is shown (rows). The presence of point mutations, CNVs and both are indicated in red, blue and green, respectively

Sixteen patients underwent ctDNA analysis before everolimus treatment. Somatic genomic alterations, including CNVs and point mutations, were detected in the ctDNA of all 16 patients (100%) (Additional file 2: Table S2). The number of somatic mutations in each patient ranged from 2 to 15, and the mean number was 7.2. The commonly mutated genes were *PIK3CA, TP53*, *ESR1*, *ERBB2* and *ALK* (Fig. 4).

**Fig. 5:**
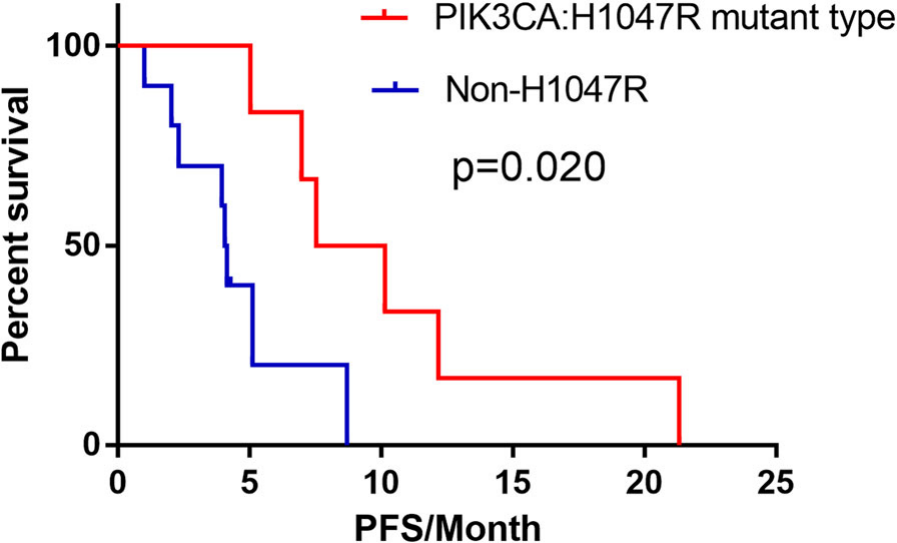
The PFS of patients treated with everolimus based on *PIK3CA*/H1047R mutations. A total of 16 patients underwent ctDNA analysis; 6 patients had *PIK3CA*/H1047R mutations (blue), and 10 patients did not have *PIK3CA*/H1047R mutations (red). PFS, progression-free survival. *PIK3CA*, phosphatidylinositol 3-kinase

*PIK3CA* gene mutations were detected in 10 (62.5%) patients and included 10 point mutations and 1 CNV. *PIK3CA/*H1047R was the most frequently detected mutation, as it appeared in 6 patients, and the other four types of mutations, namely, H1047L, H1047Y, N345K, and E545K, were each detected in 1 patient. Patients with the *PIK3CA*/H1047R mutation had longer PFS than patients with wild-type or other mutant forms of *PIK3CA*, and the median PFS was 8.8 and 4.1 months, respectively (*p* = 0.020, Fig. 5). However, we did not find similar differences in survival between patients with other types of *PIK3CA* mutations and patients with wild-type *PIK3CA* in whom the median PFS was 4.6 and 7.0 months, respectively (*p* > 0.05).

## Discussion

The resistance of endocrine therapy is a major clinical challenge. The mechanism of endocrine therapy resistance is very complex, and one of the most important mechanisms is the activation of the *mTOR* signaling pathway [4]. Everolimus, an *mTOR* inhibitor, can enhance the efficacy of endocrine therapy and may reverse drug resistance [21]. We performed the present study to find patients who will benefit most from or exhibit resistance to everolimus.

In the present study, everolimus combined with endocrine therapy in patients with HR-positive metastatic breast cancer resulted in a PFS of 5.1 months. The PFS identified in the present study is shorter than the PFS in the BOLERO-2 study [5]. This disparity can be accounted for by the retrospective design of this study and the higher tumor and treatment burdens of the patients. In the present study, 45.0% patients had > 2 metastatic sites, 81.7% patients received > 2 lines of previous endocrine therapies, and 43.0% received > 2 lines of previous chemotherapy. However, in the BOLERO-2 study, only 35% of patients had > 3 metastatic sites, and 26% received chemotherapy in metastatic settings before everolimus [5, 6]. In our study, < 3 metastatic sites, < 2 lines of previous endocrine therapy, and < 2 lines of previous chemotherapy were associated with improved survival.

Additionally, the AEs profile in this study was similar to that of previous studies [5–7, 22]. Consistent with previous reports, stomatitis was the most frequent treatment-related adverse event. However, the frequency of stomatitis in our study was lower than that reported in previous studies. This discrepancy may be due to each patient receiving an oral care package that prevented stomatitis in our study. Effective intervention and prevention may help reduce the occurrence of severe stomatitis. Kangfuxinye has been shown to have a potential preventive effect against stomatitis and was reported to be effective in the prevention of mucositis induced by chemoradiotherapy in a phase III clinical study of nasopharyngeal carcinoma [23].

Subgroup analyses in the TAMRAD study indicated that the median time to tumor progression (TTP) was 14.8 months vs 5.4 months in patients with secondary resistance and patients with primary resistance, respectively [7]. Similarly, patients with secondary resistance to AIs had a significantly higher CBR with everolimus combined with tamoxifen (74%) than with tamoxifen alone (48%) [7]. However, in the present study, no difference in survival was observed between patients who received endocrine drugs that were used in previous treatments and patients who did not use any endocrine drugs (median PFS 5.23 and 5.10 months, respectively, *p*>0.05). We also did not find any difference in outcomes between patients who received endocrine drugs to which they acquired primary resistance in previous treatments and patients who received endocrine drugs to which they acquired nonprimary resistance (*p* > 0.05). A real-world retrospective study performed in China also found results similar to ours [22].

Thus far, no study has directly compared the efficacy among different endocrine drugs combined with everolimus. In our study, multivariate analysis indicated that treatment with everolimus combined with fulvestrant was associated with improved survival. However, these results require further confirmation in large-scale randomized clinical trials.

Mutations in the *PIK3CA/AKT/mTOR* pathway are frequent in breast cancer patients [24, 25]. Experimental studies have demonstrated that breast cancers with *PIK3CA* mutations are more sensitive to everolimus, but this has not been confirmed in clinical studies [12–14]. This result may be due to the heterogeneity of the mutational status between primary tumors and metastases and the small number of patients included in previous studies [8, 26]. In the present study, the frequency of *PIK3CA* mutations was 62.5%, which was higher than that reported in previous studies (20–45%) [19, 24]. This discrepancy may have occurred because patients in the present study had advanced cancer with increased treatment burden or because *PIK3CA* mutations are significantly more frequent in HR-positive cancers than in HR-negative cancers [24, 27, 28]. Based on the present study, patients with the *PIK3CA*/H1047R mutation had longer PFS than patients with wild-type or other mutant forms of *PIK3CA*, with median PFS rates of 8.8 and 4.1 months, respectively (*p* = 0.020). However, we did not find similar differences in survival between patients with other types of *PIK3CA* mutations and patients with wild-type *PIK3CA* in whom the median PFS was 4.6 and 7.0 months, respectively (*p* > 0.05). In the BOLERO-2 study, mutational analysis of plasma cell-free DNA (cfDNA) indicated no relationship between PFS after everolimus treatment and *PIK3CA* genotypes, which was consistent with a previous analysis of tumor tissue DNA [14]. Preclinical data suggested that *PIK3CA* and *mTOR* inhibitors had a lower IC50 for H1047R than for E542K or E545K [29]. The *PIK3CA*/H1047R mutation was also reported to confer sensitivity to everolimus in early-phase clinical trials in many types of cancers [30]. Therefore, *PIK3CA* mutations including H1047R, E545K, and E542K cannot predict patient responses to everolimus. Hence, it is possible that not all of the *PIK3CA* mutations confer sensitivity to everolimus and that the H1047R mutation may be a potential biomarker of sensitivity to everolimus. One possible mechanism is that the *PIK3CA/*H1047R mutation is a stronger driver of tumor development than other types of *PIK3CA* mutations such as E542K and E545K. Therefore, patients with *PIK3CA*/H1047R mutations were more sensitive to the *mTOR* inhibitor everolimus [31]. However, these results require further confirmation in clinical trials.

*PTEN* gene loss was another biomarker that was reported to be a marker of sensitivity to *PI3K/AKT/mTOR* inhibitors in preclinical studies [15, 16]. However, clinical trials including TAMRAD and BOLERO-2 did not find any association between *PTEN* gene status and everolimus efficacy [13, 32]. In the present study, a *PTEN* gene mutation was detected in only one patient, and this patient received tamoxifen plus everolimus therapy and had SD for 5.0 months. However, based on just one patient, we were unable to find an association between *PTEN* gene mutations in ctDNA and response to everolimus.

Despite the advantages described above, several limitations of this study should be noted. First, the retrospective design and the small sample of patients who underwent ctDNA analysis do not provide sufficient power to derive statistically sound conclusions. Additionally, because of the small sample size, we did not analyze the association between everolimus and other genetic alterations such as *PTEN* gene loss and *KRAS* gene mutations, which have been reported to be associated with everolimus response.

## Conclusions

In conclusion, patients who receive more lines of chemotherapy or endocrine therapy are less likely to benefit from everolimus. Additionally, for combination with everolimus, we can also select endocrine drugs that were used or not used in previous treatments as well as drugs to which the patients had acquired primary or nonprimary resistance in previous treatments. Furthermore, the *PIK3CA*/H1047R mutation may be a potential biomarker of sensitivity to everolimus.

## Additional files

Additional file 1: **Table S1.** List of target region genes. (DOC 380 kb) (DOC 379 kb)

Additional file 2: **Table S2.** Somatic mutations identified in 16 patients. (DOC 279 kb)

## Abbreviations

CBR: Clinical benefit rate
CNVs: Copy number variants.
CR: Complete response
CT: Computed tomography
ctDNA: Circulating tumor DNA
ER: Estrogen receptor
HER2: Human epidermal growth factor receptor-2
HR: Hormone
MRI: Magnetic resonance imaging
*mTOR*: Mammalian target of rapamycin
ORR: Objective repose rate
PD: Progressive disease
PFS: Progression-free survival
PI3K: Phosphatidylinositol 3-kinase
PR: Partial response
SD: Stable disease
SNVs: Single nucleotide variants
