# Supplementary material for: Everolimus in hormone receptor-positive metastatic breast cancer: PIK3CA mutation H1047R was a potential efficacy biomarker in a retrospective study
Source: BMC Cancer. 2019 May 14;19:442. doi: 10.1186/s12885-019-5668-3 (PMC6515626; doi:10.1186/s12885-019-5668-3)
Supplement: Supplementary file 2 — Table S2. Somatic mutations identified in 16 patients. (DOC 279 kb) [file 12885_2019_5668_MOESM2_ESM.doc]

| **Additional file 2: Table S2. Somatic mutations identified in 16 patients** | | | | | | |
| --- | --- | --- | --- | --- | --- | --- |
| **Patient ID** | **Gene** | **cHGVS** | **pHGVS_ad** | **Mutation AF (%)** | **PFS (months)** | **Best efficacy** |
| P1 | PIK3CA | c.3140A>G | p.H1047R | 50.50 | 5.03 | SD |
| P1 | FGFR1 | Copy number gain | - | 63.48 | 5.03 | SD |
| P1 | TOP1 | Copy number gain | - | 44.62 | 5.03 | SD |
| P1 | TP53 | c.578A>T | p.H193L | 37.24 | 5.03 | SD |
| P1 | PTEN | c.801+1G>C | - | 2.46 | 5.03 | SD |
| P1 | SF3B1 | c.2098A>G | p.K700E | 1.09 | 5.03 | SD |
| P1 | PRKCB | c.950C>A | p.P317Q | 43.09 | 5.03 | SD |
| P1 | ROS1 | c.5743G>A | p.G1915R | 1.17 | 5.03 | SD |
| P1 | MYC | c.144G>C | p.Q48H | 1.07 | 5.03 | SD |
| P1 | GATA3 | c.925-3_925-2delCA | - | 2.24 | 5.03 | SD |
| P1 | KDM5C | c.2087C>A | p.A696D | 1.14 | 5.03 | SD |
| P2 | AR | c.1369_1371delGGC | p.G457del | 13.10 | 3.93 | PD |
| P2 | KMT2C | c.3340T>C | p.C1114R | 6.33 | 3.93 | PD |
| P2 | SF3B1 | c.2960C>A | p.P987H | 1.01 | 3.93 | PD |
| P2 | PDGFRB | c.1811G>A | p.R604H | 1.29 | 3.93 | PD |
| P2 | KLF4 | c.779C>A | p.P260Q | 1.26 | 3.93 | PD |
| P2 | CAMK2G | c.66-1G>T | - | 1.05 | 3.93 | PD |
| P2 | SUFU | c.71delC | p.P24fs*72 | 1.41 | 3.93 | PD |
| P2 | SMARCA4 | c.896C>A | p.P299H | 1.11 | 3.93 | PD |
| P2 | EPHB6 | c.493_495delCCC | p.P165del | 4.04 | 3.93 | PD |
| P2 | MYC | c.144G>C | p.Q48H | 1.06 | 3.93 | PD |
| P2 | IRS2 | c.568G>A | p.A190T | 1.19 | 3.93 | PD |
| P2 | NF1 | c.2602G>T | p.G868C | 1.19 | 3.93 | PD |
| P3 | NF1 | c.2205T>A | p.Y735* | 3.59 | 5.10 | SD |
| P3 | MED12 | c.4642C>T | p.Q1548* | 1.90 | 5.10 | SD |
| P3 | TET2 | c.4546C>T | p.R1516* | 3.43 | 5.10 | SD |
| P3 | NTRK1 | c.1954G>T | p.A652S | 49.06 | 5.10 | SD |
| P3 | WT1 | c.394_396delCCG | p.P132del | 1.05 | 5.10 | SD |
| P3 | SUZ12 | c.161_163delCCT | p.S59del | 21.82 | 5.10 | SD |
| P3 | CEBPA | c.589_590insACCCGC | p.H195_P196dup | 2.40 | 5.10 | SD |
| P3 | NTRK1 | c.1954G>T | p.A652S | 47.70 | 5.10 | SD |
| P3 | ALK | c.950G>T | p.R317I | 1.50 | 5.10 | SD |
| P3 | ESR1 | c.1613A>G | p.D538G | 46.00 | 5.10 | SD |
| P3 | EGFR | c.3286T>A | p.S1096T | 0.30 | 5.10 | SD |
| P3 | MED12 | c.5285A>G | p.K1762R | 0.60 | 5.10 | SD |
| P3 | AURKA | Copy number gain | - | 5.00 | 5.10 | SD |
| P4 | TOP1 | c.2245C>T | p.R749W | 4.30 | 4.13 | PD |
| P4 | CEBPA | c.589_590insACCCGC | p.H195_P196dup | 1.64 | 4.13 | PD |
| P5 | ERBB2 | Copy number gain | - | 0.60 | 2.03 | PD |
| P5 | FGFR1 | Copy number gain | - | 0.30 | 2.03 | PD |
| P5 | PIK3CA | Copy number gain | - | 0.50 | 2.03 | PD |
| P5 | TP53 | c.734G>T | p.G245V | 0.30 | 2.03 | PD |
| P5 | AXL | c.1280G>A | p.R427H | 0.40 | 2.03 | PD |
| P5 | ESR1 | c.1613A>G | p.D538G | 0.40 | 2.03 | PD |
| P5 | BCOR | c.1423T>G | p.L475V | 0.50 | 2.03 | PD |
| P5 | CHD2 | c.493A>Tp | p.S165C | 0.50 | 2.03 | PD |
| P5 | PRPF4OB | c.2554C>T | p.R852C | 0.80 | 2.03 | PD |
| P5 | KIT | c.356T>G | p.L119R | 0.60 | 2.03 | PD |
| P5 | IDH1 | c.481G>A | p.G161R | 0.70 | 2.03 | PD |
| P5 | LYN | c.362T>G | p.L121R | 3.20 | 2.03 | PD |
| P5 | SRC | c.1289A>G | p.K430R | 2.40 | 2.03 | PD |
| P5 | EZH2 | c.1544A>G | p.K515R | 1.20 | 2.03 | PD |
| P5 | PIK3CA | c.3139C>T | p.H1047Y | 1.10 | 2.03 | PD |
| P6 | BRAF | c.964G>A | p.A322T | 1.26 | 8.70 | SD |
| P6 | MLL | c.4778G>A | p.R1593H | 1.13 | 8.70 | SD |
| P6 | RNASEL | c.505G>T | p.A169S | 1.44 | 8.70 | SD |
| P6 | ZNF703 | c.1454C>T | p.T485M | 1.15 | 8.70 | SD |
| P6 | CREBBP | c.6761T>C | p.L2254P | 1.32 | 8.70 | SD |
| P6 | NR3C1 | c.218C>T | p.A73V | 1.08 | 8.70 | SD |
| P6 | GATA2 | c.572C>T | p.A191V | 1.27 | 8.70 | SD |
| P6 | MLL | c.11455C>T | p.R3819C | 1.04 | 8.70 | SD |
| P6 | VHL | c.430G>A | p.G144R | 1.25 | 8.70 | SD |
| P6 | EP300 | c.5705C>T | p.A1902V | 1.23 | 8.70 | SD |
| P6 | MLL3 | c.4672C>T | p.R1558W | 1.05 | 8.70 | SD |
| P7 | DNMT1 | c.2621A>G | p.D874G | 1.48 | 1.00 | PD |
| P7 | EIF4A2 | c.716A>G | p.K239R | 1.72 | 1.00 | PD |
| P7 | NFE2L2 | c.371C>T | p.A124V | 1.24 | 1.00 | PD |
| P7 | TAF1 | c.3650G>A | p.R1217H | 1.22 | 1.00 | PD |
| P7 | TOP2A | c.3595A>G | p.K1199E | 1.42 | 1.00 | PD |
| P7 | ZRSR2 | c.1355G>A | p.R452H | 1.11 | 1.00 | PD |
| P7 | MTOR | c.4286C>T | p.A1429V | 1.15 | 1.00 | PD |
| P7 | MYC | c.144G>C | p.Q48H | 1.01 | 1.00 | PD |
| P7 | ALK | c.224C>A | p.P75Q | 1.04 | 1.00 | PD |
| P7 | ZNF217 | c.1073C>T | p.A358V | 1.10 | 1.00 | PD |
| P7 | ELAC2 | c.397C>A | p.L133I | 1.03 | 1.00 | PD |
| P8 | PIK3CA | c.3140A>G | p.H1047R | 1.50 | 7.53 | SD |
| P8 | FLT3 | c.826C>A | p.H276N | 1.10 | 7.53 | SD |
| P8 | TP53 | c.817C>T | p.R273C | 0.90 | 7.53 | SD |
| P9 | HCLS1 | c.1274G>T | p.G425V | 34.50 | 21.33 | PR |
| P9 | BRD3 | c.719A>G | p.K240R | 1.40 | 21.33 | PR |
| P9 | PIK3CA | c.3140A>G | p.H1047R | 0.90 | 21.33 | PR |
| P9 | TP53 | c.783T>A | p.S261R | 0.50 | 21.33 | PR |
| P10 | ERBB4 | c.3200G>A | p.R1067Q | 1.30 | 12.17 | SD |
| P10 | PIK3CA | c.3140A>G | p.H1047R | 1.30 | 12.17 | SD |
| P10 | TERT | c.-58-u232 | 6G>A- | 1.30 | 12.17 | SD |
| P10 | MSH3 | c.72G>C | p.L24F | 1.10 | 12.17 | SD |
| P10 | PDGFRB | c.2980G>T | p.G994W | 1.10 | 12.17 | SD |
| P10 | PDGFRB | c.1323G>T | p.M441I | 1.00 | 12.17 | SD |
| P10 | NOTCH4 | c.4801C>T | p.Q1601* | 0.90 | 12.17 | SD |
| P10 | NOTCH4 | c.388A>G | p.R130G | 0.90 | 12.17 | SD |
| P10 | ESR1 | c.1138G>C | p.E380Q | 0.70 | 12.17 | SD |
| P10 | ESR1 | c.1610A>C | p.Y537S | 0.70 | 12.17 | SD |
| P10 | ESR1 | c.1613A>G | p.D538G | 0.60 | 12.17 | SD |
| P10 | ABL1 | c.2489T>C | p.L830P | 0.60 | 12.17 | SD |
| P10 | NOTCH1 | c.337A>T | p.N113Y | 0.50 | 12.17 | SD |
| P10 | MLH3 | c.1252G>T | p.E418* | 0.40 | 12.17 | SD |
| P10 | TP53 | c.733G>A | p.G245S | 0.40 | 12.17 | SD |
| P11 | PIK3CA | c.3140A>G | p.H1047R | 6.40 | 6.97 | SD |
| P11 | TP53 | c.637C>T | p.R213* | 3.40 | 6.97 | SD |
| P11 | EPHA5 | c.2737G>C | p.D913H | 1.40 | 6.97 | SD |
| P11 | CCNE1 | c.83C>G | p.S28C | 0.70 | 6.97 | SD |
| P11 | TERT | c.-58-u5148C>A | . | 0.60 | 6.97 | SD |
| P12 | EPHA3 | c.2074+1G>C | . | 9.50 | 10.13 | SD |
| P12 | PIK3CA | c.3140A>G | p.H1047R | 7.70 | 10.13 | SD |
| P12 | INPP4B | c.2743C>G | p.P915A | 5.90 | 10.13 | SD |
| P12 | MAP3K1 | c.2384dupT | p.S796Vfs*11 | 6.10 | 10.13 | SD |
| P12 | APC | c.7403C>T | p.S2468L | 7.40 | 10.13 | SD |
| P12 | FOXA1 | c.798C>G | p.F266L | 12.20 | 10.13 | SD |
| P12 | COL5A1 | c.2770C>T | p.R924W | 0.50 | 10.13 | SD |
| P12 | THOC1 | c.1107delC | p.D370Mfs*9 | 1.10 | 10.13 | SD |
| P13 | PIK3CA | c.1633G>A | p.E545K | 1.70 | 4.07 | PD |
| P13 | TP53 | c.574C>T | p.Q192* | 1.10 | 4.07 | PD |
| P13 | CDH23 | c.6511C>T | p.R2171C | 0.70 | 4.07 | PD |
| P14 | TP53 | c.708_709delCA | p.M237Vfs*2 | 6.00 | 4.20 | SD |
| P14 | FLT1 | c.1A>G | p.0? | 5.80 | 4.20 | SD |
| P14 | CDK12 | c.4130C>T | p.S1377L | 0.60 | 4.20 | SD |
| P14 | NOTCH1 | c.6395C>T | p.T2132M | 0.50 | 4.20 | SD |
| P14 | CDK12 | Copy number gain | - | 8.40 | 4.20 | SD |
| P14 | ERBB2 | Copy number gain | - | 5.00 | 4.20 | SD |
| P15 | TP53 | c.702C>A | p.Y234* | 16.80 | 2.30 | PD |
| P15 | PIK3CA | c.3140A>T | p.H1047L | 14.60 | 2.30 | PD |
| P16 | MAP3K1 | c.4151_4158delTAAGAATT | p.L1384Rfs*33 | 12.90 | 4.27 | PR |
| P16 | MLL3 | c.14247_14253delTTATAGT | p.Y4750Nfs*14 | 12.50 | 4.27 | PR |
| P16 | TBX3 | c.620_621delTG | p.L207Qfs*19 | 10.00 | 4.27 | PR |
| P16 | PIK3CA | c.1035T>A | p.N345K | 7.40 | 4.27 | PR |
| P16 | ALK | c.86C>T | p.A29V | 7.30 | 4.27 | PR |
| P16 | FOXA1 | c.749C>T | p.S250F | 5.90 | 4.27 | PR |
| P16 | GATA3 | c.1042C>T | p.L348F | 4.90 | 4.27 | PR |
| P16 | ERBB2 | c.929C>T | p.S310F | 2.30 | 4.27 | PR |
| P16 | FOXA1 | c.1193_1213delACCCGTTCTCCATCAACAACC | p.H398_N404del | 1.80 | 4.27 | PR |
| P16 | ERBB2 | c.2305G>T | p.D769Y | 1.00 | 4.27 | PR |
| Abbreviations: PFS: progression-free survival; SD:stable disease; PD: disease progression; PR: partial response | | | | | | |
|
